# Effects of the healing movie programs on post-traumatic stress syndrome, resilience, and cognitive emotional control strategies of Korean cancer survivors: a non-equivalent control group pretest–posttest design

**DOI:** 10.7586/jkbns.24.015

**Published:** 2024-08-20

**Authors:** Jeong Hyeon Kong, Seonah Lee, Mi Yang Jeon

**Affiliations:** 1 University of Gyeongnam, Gyeochang, Korea; 2 College of Nursing, Chonnam National University, Gwangju, Korea; 3 College of Nursing, Sustainable Health Research Institute, Gyeongsang National University, Jinju, Korea

**Keywords:** Cancer survivors, Mental healing, Motion pictures, Stress disorders, post-traumatic, Emotional regulation

## Abstract

**Purpose:**

This study examined the effects of the healing movie programs for cancer survivors on post-traumatic stress syndrome, resilience, and cognitive emotional control strategies, a quasi-experimental design.

**Methods:**

Participants included 39 cancer survivors registered in four healthcare centers in Gyeongsangnam-do. The intervention and control group included 19 and 20 participants, respectively. The healing program using movies developed in this study was provided to the intervention group in 10 sessions, occurring twice each week for 5 weeks and lasting 80 minutes per session. The data were collected using structured questionnaires and they were analyzed to examine differences in the test scores before and after the intervention.

**Results:**

In the intervention group, the post-traumatic stress syndrome (F = 14.97, *p* < .001) decreased significantly and the resilience (F = 19.55, *p* < .001) and adaptive cognitive emotion regulation (F = 5.11, *p* = .029) increased significantly. The difference between the intervention and control group was statistically significant in post-traumatic stress syndrome, resilience, and adaptive cognitive emotion regulation. Thus, the healing program of this study significantly reduced post-traumatic stress, and it improved both resilience and adaptive cognitive emotion regulation of cancer cancer survivors.

**Conclusion:**

The healing program is an effective psychosocial nursing intervention that helps cancer survivors feel better about their illness, and it helps them healthy transition from negative emotions to increased resilience and positive cognitive emotions.

## INTRODUCTION

The diagnosis and treatment of cancer can be a psychologically traumatic experience for many patients [1]. The effects of ambiguity during cancer treatments, the nature of the psychiatric symptoms experienced, and the ongoing and deteriorating medical condition of cancer make it a life-threatening experience. Thus, the presence of post-traumatic stress disorder (PTSD) during a diagnosis of cancer is not unusual [2]. Cancer patients develop post-traumatic stress symptoms (PTSS) including intrusive trauma-related thoughts, avoidance of trauma-related reminders, hypervigilance, and heightened arousal, at any point from diagnosis through treatment, after treatment is complete, or during recurrence of the cancer [1,3]. Cancer patients with PTSS feel sadness, fear, anger, helplessness, or hopelessness. They feel detached or estranged from other people [4]. Severe emotional distress in cancer patients results in poor compliance with treatments, an unhealthy lifestyle, and increased risk of cancer progression and death [5]. Therefore, it is important to detect and appropriately treat individuals in need of psychosocial support [6].

One of the common adaptations following a traumatic event is resilience [7]. Resilience refers to the ability to “bounce back” after trauma. Thus, after a short fallout period following a catastrophic event, an individual returns to pre-event or baseline functioning [7]. Resilience is the dynamic process of adapting well in the face of adversity, trauma, tragedy, threats or even significant sources of stress [4]. Factors contributing to resilience include the ability to maintain an optimistic outlook in life, develop a positive social support network, face one's fears, and use active coping skills [8]. Resilient individuals are able to regulate their emotions effectively to adapt to stressful situations, find something positive in even adverse situations, and even experience personal growth from distressing events [7]. On the other hand, poorly resilient individuals go through distress and confusion when they try to understand the reason that this terrible event happened to them and the meaning of the event for their world view [8]. Molina et al. [9] described resilience in the three ways: a preexisting, baseline characteristic or trait, such as demographics and personal attributes (e.g., optimism and hope); an adaptation mechanism to promote positive outcomes, such as stress management and resilience training; and a psychosocial outcome itself. Promoting resilience is a critical element of patient psychosocial care. Nurses can facilitate resilience by recognizing and promoting certain baseline characteristics and optimizing mechanisms of adaptation [9].

Adjustment to cancer is a process. Successful adjustment occurs in patients who are able to regulate their emotional distress [5]. Emotion regulation is the process of maintaining a preferred or desired internal emotional state following an external emotion-eliciting stimulus [10]. Emotion regulation is performed in an automatic or controlled manner, either unconsciously or consciously [11].

Emotion regulation strategies are categorized as engagement or disengagement strategies [5]. Engagement strategies aim to change one’s emotions or thoughts following an emotional stimulus and include acceptance, active coping, cognitive reappraisal, problem solving, and seeking instrumental support. Disengagement strategies, on the other hand, attempt to lessen the impact of an emotion-eliciting event through avoidance or escape and include cognitive or behavioral avoidance, denial, substance use, and suppression [5]. Difficulties in emotion regulation were linked to emotional distress in cancer patients [5]. Positive emotion regulation strategies were closely associated with resilience in cancer patients [11,12]. The level of resilience is positively related to the degree of actively using adaptive cognitive emotion regulation strategies.

Cinema therapy is understood to promote a metaphorical therapeutic change in the client’s inner world, the same way as metaphors is used in traditional and contemporary forms of psychotherapy. Wilson [13] mentioned that films can be used in combination with cognitive behavior therapy and family system therapy. In cognitive behavior therapy, movies like stories, myths, jokes, fables, or even dreams are used in combination with the established modalities as a supportive device for understanding maladaptive core beliefs and for cognitive restructuring. Cognitive insights tell clients what to do but affective insights give them the motivation to follow through. Behavior modification treatment can be supported by watching movies where a character demonstrates courage in the face of a challenge [13].

Previous studies have reported that cinema or movie therapy is effective in combating PTSD, anxiety disorders, and other psychological responses commonly found in individuals suffering from chronic health conditions [14,15]. In cinema therapy, clients watch film(s) relevant to issues of personal distress and dysfunction [15]. Movies often deal with universal themes, which allows clients to view their problems from a comfortable distance [16]. By watching a movie, individuals can compare themselves to the characters in the movie, think outside the box, undergo perceptual and cognitive changes, and desire to express themselves to others through the indirect experience of positive and negative emotions and the process of encouraging to talk to others [14,17]. Movies are cost-effective and can generally be used with groups composed of persons with diverse backgrounds [16]. The benefits of using movies include compliance, accessibility and availability of modality, familiarity with movies, and the enhancement of rapport among group members [16]. Previous studies reported movie therapy reduce depression in elderly cancer patients [18], and increase quality of life in women with gynecologic cancer [19]. However, many of studies about movie therapy to date have not focused on cancer survivors.

In this study, we develop a healing movie program for cancer survivors and apply it to cancer survivors to examine their effects on PTSS, resilience, and cognitive emotional regulation.

### Hypotheses

Hypotheses 1: Individuals who participates in the healing movie program (treatment group) will show lower posttraumaic stress scores than those who do not participate in the program (control group).

Hypotheses 2: The treatment group will show higher resilience score than the control group.

Hypotheses 3-1: The treatment group will show higher adaptive cognitive emotion regulation score than the control group.

Hypotheses 3-2: The treatment group will show lower maladaptive cognitive emotion regulation score than the control group.

## METHODS

### 1. Study design

A pretest-posttest, non-equivalent control group, quasi-experimental design was used in this study. Specifically, a treatment and control group were tested before and after an intervention and a faux intervention (i.e., usual care) designed not to affect the dependent variable, respectively.

### 2. Participants

Participants were recruited from four different public healthcare centers located in the Gyeongnam province, South Korea. These public healthcare centers provided the same organizational characteristics, structure, management, and service care for community dwellers. The participants eligible for this study were adult cancer survivors who were within 5 years of completing cancer treatments, maintained independence in daily activities, reported no other complications due to cancer or cancer treatments, understood the purpose of the study, consented to voluntary participation in the study, reported no previous experience with movie therapy, and they were registered at a healthcare center. Exclusion criteria included receiving cancer treatments over 5 years before the start of the study and difficulty in communication.

Sample size was calculated using the G*Power 3.1 program [20]. A one-tailed t-test with statistical power (1-β) = .80, a significance level (α) = .05, and effect size (ɖ) = .80 showed that each group required at least 16 participants. Forty-two participants including 21 for the experimental group and 21 for the control group were recruited. Of the four healthcare centers, two centers were conveniently assigned to the intervention group and the other two were assigned to the control group. To avoid a diffusion of the intervention, the distance between the healthcare centers for the intervention group and the healthcare centers for the control group was an hour’s drive. Two persons from the intervention group withdrew from the study due to personal reasons, and one person in the control group was excluded due to incomplete responses on the questionnaire. A total of 39 participants including 19 individuals in the intervention group and 20 individuals in the control group were included in the final sample of the study.

### 3. Instruments

#### 1) PTSS

The Impact of Event Scale-Revised Korean version (IES-R-K), which is a modified version of the Impact of Event Scale (IES) developed by Horowitz, Wilner, and Alvarez [21], was used to assess PTSS. The IES was revised by Eun et al. [22]. This instrument consists of 22 items on post-traumatic symptoms (six items for hyperarousal, six for avoidance, five for intrusion, and five for dissociation symptoms) that are rated on a 5-point scale (0 = never, 1 = rarely, 2 = sometimes, 3 = often, and 4 = always). The score ranges from 0 to 88 points. High scores indicate a high level of post-traumatic symptoms. The Cronbach's α of the IES-R-K was .83 at the time of revision by Eun et al. [22], and it was .87 in this study.

#### 2) Resilience

The Resilience scale developed by Wagnild and Young [23] and translated and revised by Song [24] was used to measure resilience. This instrument consists of 25 items including 17 items about personal competence and eight about acceptance of self and life. The items are rated on a 5-point scale (1 = never, 2 = rarely, 3 = sometimes, 4 = often, and 5 = always). The scores range from 25 to 125 points. High scores indicate a high level of resilience. The Cronbach’s α of the instrument was .88 in a study by Song [24], and it was .84 in this study.

#### 3) Cognitive emotion regulation

The modified version of the Cognitive Emotion Regulation Questionnaire (CERQ) developed by Garnefski, Kraaij, and Spinhoven [25], namely, the CERQ-Korean version (K-CERQ) [26] was used to assess cognitive emotion regulation. This instrument contains 36 questions rated on a 5-point scale (1 = strongly disagree, 2 = disagree, 3 = neutral, 4 = agree, and 5 = strongly agree). The questionnaire includes 20 items about adaptive cognitive emotion regulation and 16 items about maladaptive cognitive emotion regulation. The scores of adaptive cognitive emotion regulation range from 20 to 100 points. High scores indicate frequent use of adaptive cognitive emotion regulation strategies. The scores of maladaptive cognitive emotion regulation range from 16 to 80 points. High scores indicate frequent use of maladaptive cognitive emotion regulation strategies. At the time of instrument development, the overall Cronbach's α for the tool was .93, the adaptive cognitive emotional regulation was .91, and the maladaptive cognitive emotional regulation was .87 [25]. In this study, the overall Cronbach's α was .84, the adaptive cognitive emotional regulation was .92, and the maladaptive cognitive emotional regulation was .80.

### 4. Development of the healing movie program

#### 1) Intervention need assessment

To assess the needs of an intervention to improve quality of life for cancer survivors, five cancer survivors were interviewed. Interview questions: What is your experience like as a cancer survivor? What programs are available to help you live as a cancer survivor?. They remembered cancer experiences as a terrible crisis characterized by negative emotions and physical distress although they completed all cancer treatments. Thus, it was necessary to provide a nursing intervention to alleviate cancer survivors’ suffering.

#### 2) Training for the healing movie therapy

The researcher (Kong JH) participated in the Healing Cinema course and received the certificate of a cinema specialist. The researcher completed the six-credit course of psychological trauma and care in a graduate school and finished the 8-hour PTSS course provided by the Korean Counseling Psychological Association. She also had many educational experiences involving stress management for patients with chronic diseases registered at healthcare centers.

#### 3) Development of the healing movie program

The healing movie program using movies was developed based on the findings of previous studies on movie therapy [18,26]. The mean time of program implementation was 8.4 weeks and the program ran for 9.1 sessions on an average. Programs using visual media ran for 8–10 sessions on average [27,28]. We decided to use 10 sessions, at 80 minutes per session, conducted twice each week for a duration of 5 weeks (Table 1). We chose movies accessible and familiar to any cancer survivors (Table 1).

The healing movie program developed was used as the intervention to help the participants decrease their negative and painful emotions and reconstitute a healthy life. As the participants observe behaviors and emotions of main characters of movies, connect with main characters and themselves, and expose their suppressed emotions in conversation with other cancer survivors, it is anticipated that they address PTSS, improve resilience, increase adaptive cognitive emotion regulation, and decrease maladaptive cognitive emotion regulation. The program was composed of three parts, including the introduction, deployment, and ending (Table 1).

#### 4) Content validity of the healing movie program

Three experts were asked to assess the content validity of the healing movie program developed. They included a professor who was an oncologists, a movie therapist, and a mental health nurse. The content validity was 0.90 indicating an acceptable level [29].

### 5. Procedures for the intervention group and the control group

The healing movie program was provided in two healthcare centers assigned to the intervention group. The intervention groups included 10 and 11 participants registered in each healthcare center. The researcher (Kong JH) provided the intervention groups with the healing movie program at scheduled dates and times.

At the beginning of each session, the researcher introduced the movie content, the activities that the participants would engage in after watching the movie, and the estimated duration of each activity. The participants watched a movie and had a conversation about a movie and their feelings, immediately followed by relaxation therapies and scheduled activities facilitating the main intervention program (Table 1).

The control group received usual care providing a pamphlet about the healthy lifestyle for cancer survivors. Providing the pamphlet is a routine of the healthcare centers of the control group. For the ethical reasons, the researcher provided the control group with one session of the healing movie program after finishing all of the sessions for the intervention groups.

### 6. Intervention

In each session except for the first and last session, the participants watched a movie for 10 to 15 minutes and then talked about the movie and their feelings for 20 to 15 minutes, which was a total of 40 minutes (Table 1). Relaxation therapy, such as deep breathing, muscle relaxation, laughing, forgiveness meditation, and happiness meditation, lasted for 10 minutes in each session. Scheduled activity, such as telling personal stories when he or she was happy, writing thank-you letters, drawing a graph of his or her life facing challenges, planning positive activities, imaging his or her happy life after a year, practicing forgiveness, practicing finding sources of happiness in daily life, and practicing smiling, was implemented for 30 minutes in each session. Relaxation therapy and scheduled activities were employed to facilitate and assist the healing movie program.

### 7. Data collection

**Figure 1.**
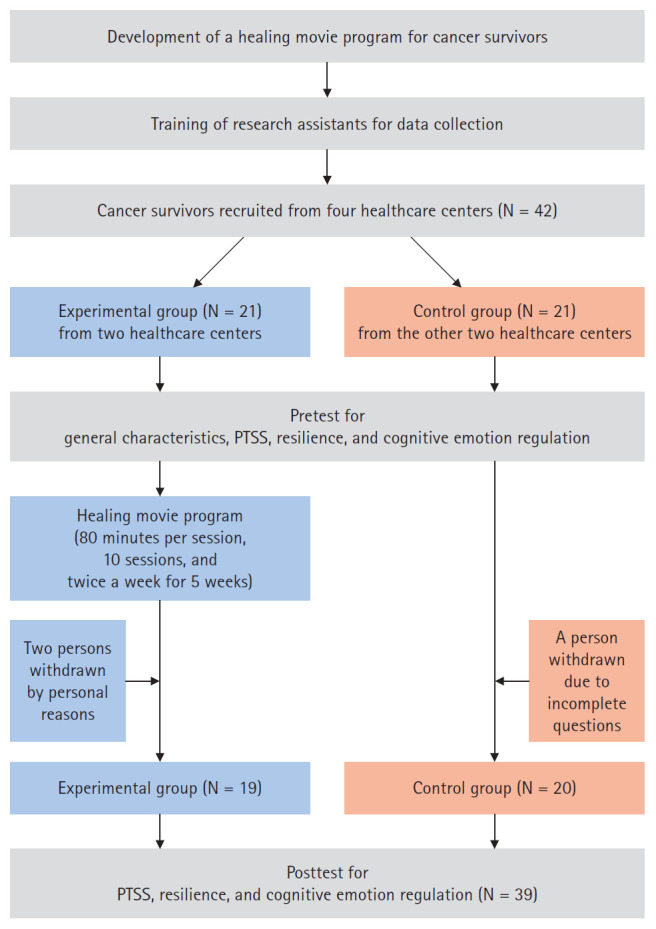
A flowchart of the study. PTSS = Post-traumatic stress syndrome.

Two graduate students in a master program of nursing collected the pretest and posttest data for the two groups. The researcher informed the graduate students about brief information on the study and surveying. The graduate students conducted a pretest for the intervention group before the first class of the implementation of the healing movie program and a posttest within a week after the last class of the program at both the healthcare centers. The participants read and completed the questionnaires by themselves. The data for the control group were collected at the control group’s healthcare centers within a week of the program started and finished, for the pretest and posttest respectively (Figure 1).

### 8. Statistical analysis

The collected data were analyzed using IBM SPSS Statistics software (version 25.0; IBM., Armonk, NY, USA) for Windows. Subjects’ general characteristics were analyzed in terms of frequency and percentages, or the mean and standard deviation. The reliability of the instruments was analyzed using Cronbach’s α. Before the intervention, the homogeneity of the intervention and control group was analyzed using chi-square test and Fisher’s exact test and independent samples t-test. We examined the effects of the intervention using ANCOVA. The homogeneity between groups on the pretest results of the research variables was confirmed, but covariance analysis using the pretest score as a covariate was conducted to test the effectiveness of the program.

### 9. Ethical considerations

We obtained the approval for this study from the institutional review board at Gyeongsang National University, South Korea (approval no. GIRB-A14-W-0037). All participants were informed about the purpose of the study, the procedures of data collection, the provision of intervention, their rights of participation, and confidentiality and anonymity of the data collected. All participants provided written informed consent before the pretest.

## RESULTS

### 1. A homogeneity test for general characteristics

The test assessing the homogeneity of the general characteristics of the participants and research variables (i.e., PTSS, resilience, adaptive and maladaptive cognitive emotion regulation) showed no significant differences between the intervention and control group at the baseline (Table 2, 3).

In the intervention group, the mean age of the participants was 64.53 ± 6.86 years. Appoximately 37% of the participants were males, 89.5% had religion, and 63.2% were married. In the control group, the mean age of the participants was 63.80 ± 6.69 years. Forty percentage of the participants were males, 90% had religion, and 75% were married.

### 2. Effects of the healing movie program

Hypothesis 1: The PTSS in the intervention group were significantly lower at post-intervention compared with pre-intervention (t = 4.98, *p* < .001), whereas the score change in the control group was not statistically significant. When we tested the effects of the program, the intervention group showed a significantly lower score in PTSS compared with the control group (F = 14.97, *p* < .001).

Hypothesis 2: Resilience in the intervention group were significantly higher at post-intervention compared with pre-intervention (t = -4.48, *p* < .001), whereas the score change in score in the control group was not statistically significant. The ANCOVA revealed that the intervention group showed a significantly higher score in resilience compared with the control group (F = 19.55, *p* < .001).

Hypothesis 3-1: The change in adaptive cognitive emotion regulation was not significant in both the intervention group (t = -2.06, *p* = .051) and the control group (t = 1.79, *p* = .087), whereas the ANCOVA showed the significant effect of interaction of time and group (F = 5.110, *p* = .029).

Hypothesis 3-2: The change of maladaptive cognitive emotion regulation was not significant in the intervention group (t = 1.23, *p* = .232), but in the control group (t = -2.11, *p* = .047), it was significant. The ANOVA showed no significant effort of interaction of time and group (F = 2.75, *p* = .105).

## DISCUSSION

This study investigated the effects of the healing movie program developed for cancer survivors. The program included watching a movie, conversation about a movie and the participants’ feelings, and various activities such as telling, writing, drawing, imaging, forgiving, and smiling. Through the healing movie program, cancer survivors became more aware of their negative emotions, expressed their feelings in a conversation session, and developed a positive mindset. Accordingly, this engagement reduced post-traumatic stress, and increased resilience and positive adaptive cognite emotion regulation in the statistically significant levels.

The PTSS score of the intervention group significantly decreased after the intervention of the healing movie program. The healing movie therapy encouraged the participants to freely talk to other participants about their feelings regarding the movie and express their own emotions recalled by the movie. The participants expressed their long-suppressed feelings to other participants in a relaxed manner. This exposure helped the participants reduce their tendency to avoid traumatic memories associated with cancer diagnosis and treatment in a negative manner. Frueh [30] reported a significant decrease of PTSS in Vietnam veterans by using exposure therapy that included a variety of means, such as reading and talking about the war, reflecting on traumatic war experiences, and sitting near a helicopter pad as well as watching Vietnam war movies. Self-exposure was a useful way for patients who experienced traumatic events to resolve their emotional issues [31]. Based on the results of this study and previous studies, it is thought that exposing cancer survivors' emotions while watching movies in this study reduced PTSS in cancer survivors who experienced trauma events.

The resilience score of the experimental group significantly increased after the healing movie program intervention. The results of this study were consistent with the results of Loprinzi et al. [32] that showed that individual resilience could be improved by training. In their study, breast cancer survivors’ resilience significantly increased at the end of a 12-week training program that focused on attention and interpretation to decrease stress and enhance resilience. To enhance the resilience of cancer survivors, our program included various activities such as filling one’s self with happiness, writing thank-you letters, developing a graph of his or her life facing challenges, planning positive activities, imagining a happy life after 1 year, practicing forgiveness, finding sources of happiness in daily life, planning a happy and healthy future, and practicing smiling. Through these activities, the participants promoted a positive mindset and realized that happiness is not beyond their reach, and eventually promoted resilience. Moreover, by forgiving those who hurt them in the past and ending unhealthy relationships with them, the participants felt at ease, took a load off their mind, gained positive emotions of self-satisfaction and happiness and thus, increased their resilience.

The score of the adaptive cognitive emotion regulation of the intervention group increased after the intervention. The effects of this intervention lasted for 2 weeks. Adaptive cognitive emotion regulation plays a key role in individual’s growth by encouraging positive refocusing and reevaluation, wider perspectives, review of plans, and acceptance, and by contributing to the reconstruction of the existing belief systems [25]. In our program, by watching movies of protagonists with cancer and listening to their stories, the participants stopped blaming themselves for causing cancer and repeating negative thoughts from their past experiences. The program encouraged the participants to explore ways to positively change their current situation through the protagonists of movies who courageously challenged themselves. Thus, the program contributed to the improvement of adaptive cognitive emotion regulation such as positive refocusing and reevaluation.

The score of the maladaptive cognitive emotion regulation of the intervention group decreased after the intervention. Previous studies presented a decrease in maladaptive cognitive regulation after the similar intervention. Massah et al. [33] reported that the emotion regulation strategies of their training program reduced anger symptoms of drug-dependent individuals. As one type of maladaptive cognitive emotion regulation, cancer survivors tend to blame themselves or other individuals for developing cancer. According to the study by Park and Kim [34], when asked about the factors that caused their cancer, female cancer survivors blamed other individuals, such as their spouse and mother-in-law, whereas male cancer survivors blamed themselves for not taking care of their own health. In this study, participants had time to share their feelings with other participants while watching a movie to reduce maladaptive cognitive emotion regulation. The participants expressed emotions, such as the fear and horror of recurrence, guilt, and depression. However, they did not fully express their own feelings or feelings toward people who were associated with causing their cancer. This means that it is necessary to develop intervention strategy to reduce negative emotion in cancer survivors.

This study had several limitations. First, because the participants were recruited from four healthcare centers, the findings of this study were limited to this population. Second, the participants who enrolled in the intervention program of this study might be highly motivated to learn and practice the skills provided in the program. This might influence the results of this study. Third, all the participants were over 50s although the researchers did not intend to recruit those participants. Studies involving participants of various ages are needed. Forth, follow-up tests were not planned and conducted to test the lasting effects of the healing movie program. The effectiveness of movie therapy requires validation with a randomized control group with long term follow-up tests. Thus, the results of this study provide the preliminary evidence of the effectiveness of an audiovisual intervention, such as movie therapy. Lastly, the skill set of the researcher (Kong JH) who conducted the healing movie program with the intervention group might play a role in the positive results of this study. Future program results can be affected by the skill set of the movie therapists who conduct such programs and this should always be considered when studying the use of such programs.

## CONCLUSION

In this study, the healing movie program for cancer survivors was developed and applied for 5 weeks to confirm the effect on changes in PTSS, resilience, and cognitive emotion regulation strategies. The healing movie program developed in this study was found to have the effect of reducing PTSS and increasing resilience by allowing cancer survivors to express their emotions in words or in writing through the experience of the protagonist in the movie.

Since the above research results have confirmed that the healing movie program has a positive effect on the PTSS and resilience of cancer survivors, it is suggested to use it as a community program for cancer survivors who have experienced and recovered from cancer. As a suggestion for further research, first, based on cognitive behavior theory, it is suggested to develop a program that not only promotes the adaptation to the life of cancer survivors but also returns to the community and second, to develop customized health programs focusing on specific cancers and to identify their effects.

## Figures and Tables

**Table 1.** Provision of the Healing Movie Program

Session	Topic	Movie’s name	Activities after watching a movie	Time (min)
Introduction				
1	Happy meeting		• Self-introduction and making a nickname	60
Deployment				
2	Pursuit of happiness	Family ties	• Talk about emotional obstacles to your life	40
			• Filling myself with happiness: tell 1-3 stories	10
				30
3	Courage	Fly, penguin	• Talk about courage and emancipation from refusal	40
			• Encourage	10
			• Write thank-you letters to those you appreciate	10
				20
4	Challenge	My granny is in 1^st^ grade	• Talk about emancipation from failure and challenge	40
			• Draw a graph of my life facing challenges	10
				30
5	Anger control	KBS special documentary ‘Mind’ episode 1	• Talk about emancipation from and control of anger	40
			• Plan positive activities	10
				30
6	Self-esteem	Miss Granny	• Talk about emancipation from acknowledgement and self-esteem	40
				10
				30
7	Forgiveness	KBS special documentary ‘Mind’ episode 6	• Talk about emancipation from negativity and forgiveness	40
			• Practice forgiving	10
				30
8	Increasing a feeling of happiness	MBC special documentary ‘Living in the Jiri mountain’	• Talk about enjoying daily life and enhancement of happiness	40
			• Practice finding sources of happiness in daily life	10
				30
9	Increasing a feeling of happiness	Lecture 100°C – a doctor who overcame cancer. Hwang Youngjae	• Talk about puissance of affirmation that makes a miracle	40
		‘Positive energy makes a miracle’	• Practice smiling	10
				30
Ending				
10	Enhancing happiness		• Look at myself before and after the program	40
			• Plan and share a happy and healthy future	10
			• Completion ceremony, and making a promise to practice my change	30

KBS = Korean Broadcasting System; MBC = Munwha Broadcasting Corporation.

**Table 2.** A Homogeneity Test of the General Characteristics and Research Variables (N = 39)

Variables	Characteristics	Exp. group (n = 19)	Con. group (n = 20)	*χ*^2^/t/Z	*p*
		n (%)/ M ± SD			
Sex	Male	7 (36.9)	8 (40.0)	0.04	.839
	Female	12 (63.2)	12 (60.0)		
Age^†^(yr)	50∼< 60	5 (26.3)	3 (15.0)	1.39	.533
	60∼< 70	8 (42.1)	12 (60.0)		
	70∼< 75	6 (31.6)	5 (25.0)		
Religion	Yes	17 (89.5)	18 (90.0)	0.65	.878
	No	2 (10.5)	2 (10.0)		
Spouse	Yes	12 (63.1)	15 (75.0)	0.64	.423
	No	7 (36.9)	5 (25.0)		
Economic state	Difficult	11 (57.9)	10 (50.0)	0.24	.621
	Not difficult	8 (42.1)	10 (50.0)		
Cancer diagnosis^†^	Gastric cancer	5 (26.3)	4 (20.0)	1.11	.831
	Breast cancer	6 (31.5)	5 (25.0)		
	Thyroid cancer	4 (21.1)	4 (20.0)		
	Other cancer	4 (21.1)	7 (35.0)		
A period of cancer diagnosis (yr)	1∼< 2	7 (36.9)	4 (20.0)	2.69	.333
	2∼< 3	2 (10.5)	6 (30.0)		
	3∼4	10 (52.6)	10 (50.0)		
Seriousness of being diagnosed with cancer^†^	Not	6 (31.5)	3 (15.0)	1.56	.490
	A little	9 (47.4)	11 (55.0)		
	A lot of	4 (21.1)	6 (30.0)		
Uncomfortable symptoms by cancer treatments^†^	Not	5 (26.3)	4 (20.0)	0.27	<.999
	A little	10 (52.6)	12 (60.0)		
	A lot of	4 (21.1)	4 (20.0)		
Difficulty in daily life by cancer treatments^†^	Not	2 (10.5)	2 (10.0)	0.20	<.999
	A little	13 (68.4)	14 (70.0)		
	A lot of	4 (21.1)	4 (20.0)		
PTSS		41.96 ± 6.94	42.04 ± 11.64	-0.03	.976
Resilience		77.09 ± 12.71	77.08 ± 8.95	-0.46	.621
Cognitive emotion regulation	Adaptive	69.13 ± 10.30	69.87 ± 9.28	0.26	.799
	Maladaptive	47.83 ± 7.36	49.57 ± 6.16	0.87	.390

Exp. = Experimental; Con. = Control; M = Mean; SD = Standard deviation; PTSS = Post-traumatic stress syndrome.

† Fisher's Exact Test.

**Table 3.** Effects of the Healing Movie Program on PTSS, Resilience, and Cognitive Emotion Regulation

Variables	Group	Pretest	Post-test	t (*p*)	ANCOVA^†^		
		M ± SD	M ± SD		Adjusted	F	*p*
					M ± SD		
PTSS^‡^	Exp. (n = 19)	41.96 ± 6.94	30.35 ± 15.51	4.98 (< .001)	2.34 ± 0.13	14.97	< .001
	Con. (n = 20)	42.04 ± 11.64	42.04 ± 11.60	-	2.94 ± 0.13		
Resilience	Exp. (n = 19)	77.09 ± 12.71	85.61 ± 11.31	-4.48 (< .001)	85.61 ± 1.44	19.55	< .001
	Con. (n = 20)	77.08 ± 8.95	76.61 ± 13.03	0.40 (.691)	73.41 ± 1.44		
Cognitive emotion regulation							
Adaptive	Exp. (n = 19)	69.13 ± 10.30	71.43 ± 11.01	-2.06 (.051)	70.82 ± 1.03	5.11	.029
	Con. (n = 20)	69.87 ± 9.28	68.35 ± 9.51	1.79 (.087)	67.65 ± 1.03		
Maladaptive	Exp. (n = 19)	47.83 ± 7.36	46.61 ± 8.39	1.23 (.232)	47.12 ± 0.90	2.75	.105
	Con. (n = 20)	49.57 ± 6.16	50.35 ± 6.11	-2.11 (.047)	49.23 ± 0.90		

PTSS = Post-traumatic stress syndrom; ANCOVA = Analysis of covariance; M = Mean; SD = Standard deviation; Exp. = Experimental; Con. = Control.

^†^ANCOVA (pre-score was covariate); ^‡^Since the standard error of the difference between the pre and post values ​​is 0, the t value is not calculated.

## Data Availability

Please contact the corresponding author for data availability.
